# Total Pancreatectomy for Ampullary Adenocarcinoma in a 74-Year-Old Patient: Case Report and Literature Review

**DOI:** 10.1155/2020/8879609

**Published:** 2020-09-29

**Authors:** Gabriela A. Arroyo Murillo, Eleonore Choppin De Janvry, Manuela Garofalo, Fatima Della Pietra

**Affiliations:** ^1^General Surgery and Organ Transplantation Unit, Department of Surgery, University of Rome La Sapienza, Rome, Italy; ^2^General Surgery and Bariatric Surgery Unit, Department of Surgery, Civita Castellana Hospital, Civita Castellana, Italy

## Abstract

Primary ampullary neoplasms have origin in the ampulla of Vater, an anatomical structure where the common bile duct and the pancreatic duct join together as a common channel. It represents <0.5% of all gastrointestinal cancers and approximately 7% of all periampullary cancers. The adenocarcinomas arising in this region originate from different epithelial cellular constituents present at the site, the histopathological classification encompass: intestinal type, pancreaticobiliary type, and mixed type. Pancreaticoduodenectomy is the treatment of choice when there is an overt or highly suspicious malignant behaviour. We present here the case of a 74-year-old male patient who presented to our department for further investigation of obstructive jaundice and a pancreatic mass associated with a six-month history of significant weight loss and mild epigastric pain. Eventually, a total splenopancreatectomy was performed given the extension of structural anomalies of the organ secondary to an ampullary adenocarcinoma.

## 1. Introduction

Primary ampullary neoplasms have origin in the ampulla of Vater, an anatomical structure where the common bile duct and the pancreatic duct join together as a common channel. It represents <0.5% of all gastrointestinal cancers and approximately 7% of all periampullary cancers. [1]

The adenocarcinomas arising in this region originate from different epithelial cellular constituents present at the site, the histopathological classification encompass: intestinal type, pancreaticobiliary type, and mixed type. [2]

Carcinomas of the pancreaticobiliary subtype are found to be more aggressive than that of the intestinal subtype (5-year disease survival rate of 48% vs. 73% [3]). In other studies, however, such an association has not been observed [4].

Immunohistochemistry and genomic aspects of ampullary cancers are considered relevant matters of discussion [2, 5] and may have prognostic implications, as well as influencing the efficacy of chemotherapy [6].

According to the model proposed by Ang et al., immunohistochemical (IHC) stains may allow distinction between the histologic subtypes. In particular, tumors that stain positive for CK-20 (cytokeratin 20), CDX2 (caudal type homeobox 2), or MUC2 (mucin-2) in the absence of MUC1 (mucin-1) staining, or positive for all of the first three regardless of MUC1, are classified as intestinal. Tumors that stain positive for MUC1 and negative for CDX2 and MUC2 are classified as pancreaticobiliary [5].

The management of ampullary neoplasms depends on multiple factors; pancreaticoduodenectomy is the treatment of choice when there is an overt or highly suspicious malignant behaviour. The rate of potentially curative resection is as high as 90%, and in high-volume centres, an acceptable rate of complications is reported. Endoscopic ampullectomy is the gold standard in case of low- up to high-grade dysplasia providing a proper assessment of the *T* status by endoscopic ultrasound. [7]

## 2. Case Presentation

### 2.1. History

A Caucasian 74-year-old male patient was referred to our hospital for further investigation of a pancreatic mass associated with a six-month history of significant weight loss and mild epigastric pain. Past history included obesity, obstructive sleep apnea syndrome, atrial fibrillation, and previous transient ischemic attack. He denied alcohol consumption. His drug history included furosemide, digoxin, dabigatran, perindopril/amlodipine, bisoprolol, allopurinol, and simvastatin.

**Figure 1:**
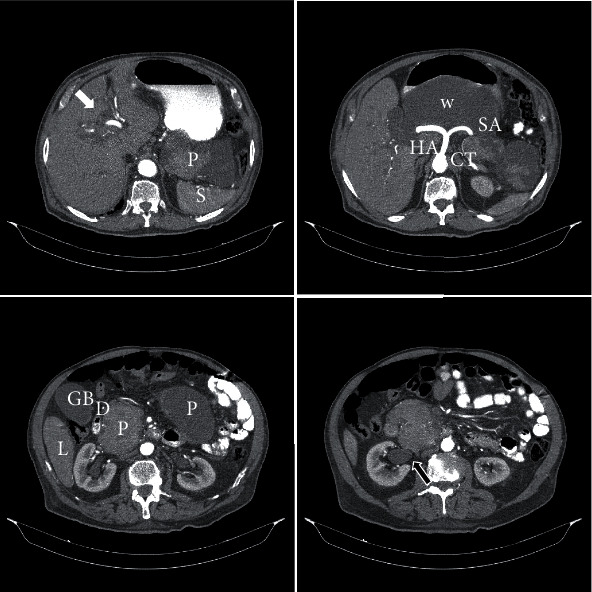
Preoperative CT scan. White arrow: biliary tree dilatation, Black arrow: right hydronephrosis. CT: celiac trunk; D: duodenum; GB: gallbladder; HA: hepatic artery; L: liver; P: pancreas; S: spleen; SA: splenic artery; W: Wirsung duct.

After the sudden appearance of obstructive jaundice, the patient was evaluated in a regional hospital without final diagnosis. A study with computed tomography (CT) scan of the abdomen showed biliary system dilatation associated with a huge cystic pancreatic mass adjacent to the right kidney and right hydronephrosis (Figure 1). A subsequent endoscopic retrograde cholangiopancreatography (ERCP) confirmed biliary tree dilatation; pancreatic duct system was anomalous, and pseudocysts were suspected during the exam. Eventually, sphincterotomy and placement of a biliary stent were performed. In that occasion, the ampulla of Vater appeared enlarged, but malignancy was not suspected, and biopsies were not performed.

On admission to our department, clinical findings were jaundice, dark urine, clay-colored stools, and itching; physical examination revealed a huge palpable mass in the epi-mesogastric region. Although urgent endoscopic decompression of the biliary tree was previously performed, the results of laboratory exams showed hyperbilirubinemia (20.5 mg/dL serum total bilirubin with 18 mg/dL of conjugated bilirubin); AST: 86 IU/L; ALT: 70 IU/L; GGT: 143 IU/L; alkaline phosphatase: 427 IU/L; amylase: 10 IU/L; lipase: 14 U/L; glucose: 106 mg/dL; and CA 19.9: 15 ng/mL.

Six days after the ERCP, a cholangio-magnetic resonance showed a reduced intrahepatic and extrahepatic biliary duct dilatation; the replacement of pancreatic parenchyma by apparent pseudocysts and features of chronic pancreatitis in the remaining tissue, a pancreatic cystic neoplasm was suspected. A whole-body CT scan for staging excluded metastatic lesions, and total pancreatectomy was planned.

### 2.2. Surgical Technique

**Figure 2:**
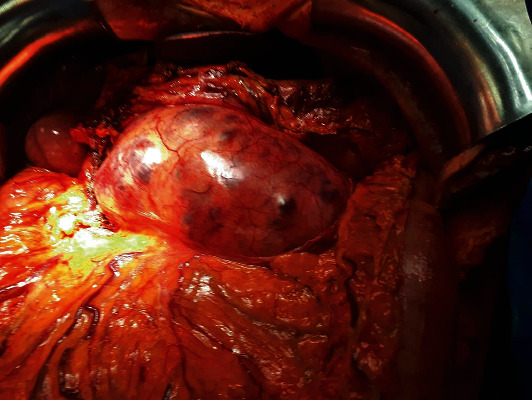
Operative field after the section of gastrocolic ligament.

A bilateral subcostal, straight transverse incision was chosen. Inspection of the peritoneal cavity excluded metastatic deposits. The lesser sac was entered after the division of the gastrocolic omentum; the right gastroepiploic pedicle was exposed and divided, and a large multicystic mass from the head to the tail of the pancreas was visualized. The voluminous tumor pushed anteriorly the gastric posterior wall and determined surgical planes alteration with caudal dislocation of transverse mesocolon (Figure 2).

**Figure 3:**
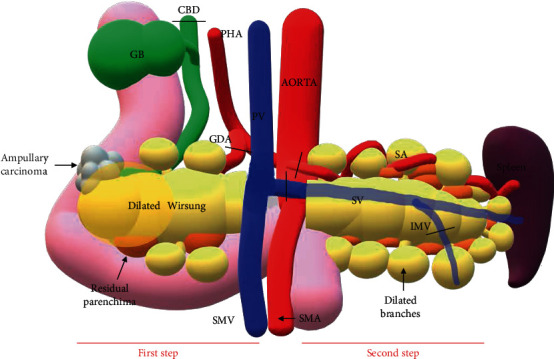
Schematic representation of the two-step procedure. GDA: gastroduodenal artery; CBD: common bile duct; GB: gallbladder; IMV: inferior mesenteric vein; PHA: proper hepatic artery; PV: portal vein; SV: splenic vein; SA: splenic artery; SMV: superior mesenteric vein; SMA: superior mesenteric artery.

Initially, hepatic flexure, distal transverse colon, and splenic flexure were mobilized; the procedure was performed in two steps considering the impact of tumor size on high-risk surgical maneuvers (Figure 3). Firstly, a pancreatoduodenectomy and later a splenopancreatectomy, the latter was performed after the section of pancreatic neck. Considering the characteristics of the lesion and splenic hilar vessels involvement by the cysts, a splenectomy was indicated.

Kocherization of the duodenum and the head of the pancreas containing the fluid-cystic lesions was performed; the aspiration of cystic fluid was necessary to complete the dissection. Inferior vena cava and left renal vein were exposed.

Superior mesenteric vein and artery were not infiltrated by the mass. Fine dissection was initiated between the pancreas and major vessels supplying the liver in order to investigate curative resectability. After cholecystectomy, common bile duct was divided proximal to the cystic duct, and gastroduodenal artery was identified and divided.

A retropancreatic tunnel above the portal vein was created, and the uncinate process was dissected over the superior mesenteric vein and superior mesenteric artery. At this point, the pancreatic neck was divided. The distal 4 cm of the stomach was transected to avoid a congested stump, and the proximal 10–15 cm of the jejunum was dissected and resected.

A meticulous mobilization of the mass allowed a hemostatically secure dissection of the mesenteric-portal vein axis until the inferior mesenteric vein was identified, dissected, and divided. Dissection of the mass from the posterior wall of the abdomen was completed.

Digestive tract reconstruction included an end-to-side hepaticojejunostomy, an antecolic end-to-side gastro-jejunal anastomosis, and a Braun anastomosis between the afferent and efferent limb distal to the gastroenterostomy.

Three drainage tubes were left in the vicinity of hepaticojejunostomy, gastro-jejunostomy, and in the splenic lodge, respectively. The intensive care unit length of stay was 2 days; the patient was initially placed on an insulin drip, and when oral diet was tolerated, he was then transitioned to a long-acting injectable insulin glargine in addition to a sliding scale of short-acting injectable insulin. Oral diet was reintroduced in the seventh postoperative day; he required a high dose of pancreatic enzyme supplements. Postoperative ascites, secondary to heart disease, responsive to medical therapy, prolonged the hospitalization. The patient was discharged in good condition on the 26th postoperative day.

**Figure 4:**
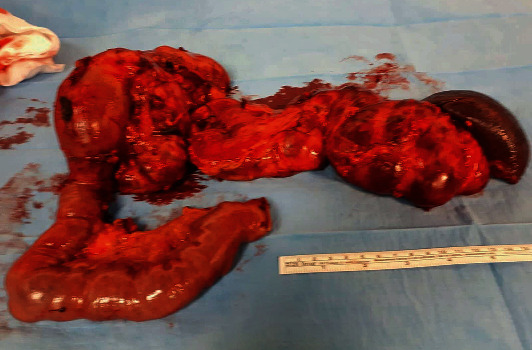
The specimen.

Pathologic examination of the specimen revealed a submucosa elevation at the level of the ampulla (3.5 cm in diameter) and a diffuse “cystic” dilatation of the main pancreatic duct and of the secondary branches caused by the ampullary tumor. Aspects of chronic pancreatitis and foci of endocrine tissue were described in a small portion of the specimen (Figure 4).

The histopathological exam showed a moderately differentiated (G2) ampullary adenocarcinoma with negative margins, and not one of the 19 lymph nodes contained tumor (pT2, N0, M0); lymphovascular and perineural invasion were not present. The immunophenotype was identified: CK7+, CK19+, CDX2+, E-caderin+, beta-catenin+ (membranous), p53+ (sporadic) CK20-, CEA-, S100-, CD10-, chromogranin A-, and synatophysin-.

## 3. Discussion

Adenocarcinomas in the periampullary region can arise from the duodenum, ampulla of Vater, distal CBD, or pancreatic duct. Importantly, different TNM stagings are applied to each of these distinct tumors [8]. We focused on the treatment of a singular case of ampullary adenocarcinoma in a geriatric patient. Currently, three approaches for the treatment of ampullary neoplasms are available: endoscopic papillectomy (EP), transduodenal ampullectomy (TDA), and pancreaticoduodenectomy (PD) [9]. None of these procedures would be eligible for our patient.

The case we reported is characterised by several peculiarities; the first one is the fact that preoperative exams led to a suspected diagnosis of pancreatic cystic neoplasm rather than ampullary adenocarcinoma. Therefore, a total pancreatectomy and lymphadenectomy was planned given the extensive morphological alterations of the pancreas.

Actually, the permanent pancreatic duct system dilatation was due to the obstruction determined by the ampullary tumor, but this was confirmed only by the pathological exam.

Another key factor to consider is the “future pancreas remnant,” its anatomical disposition, and its residual endocrine and exocrine function. In particular, small portions of residual parenchyma were identified; they corresponded to multiple foci of chronic pancreatitis containing also pancreatic islets which provided glycemic control to the patient. The patient would benefit from the metabolic advantages of a pancreatoduodenectomy, but, if a definite preoperative diagnosis of ampullary carcinoma had been made, it would have not still been possible to perform such a procedure. A pancreatojejunal anastomosis would not be feasible given the absence of an intact pancreatic stump which could ensure the tightness of the anastomosis.

We believe it is of paramount importance to highlight the necessity to carry on the procedure in two phases, because a remarkable volume of the pancreas represents a risk factor for hemorrhagic complications during surgery, especially during the mesenteric-portal vein axis dissection.

In literature, we have found only two cases of total pancreatectomy for ampullary cancer.

Savari et al. described a total pancreatectomy with islet transplantation (TPIAT) in a patient with a small, early stage ampullary cancer. In that case, an endoscopic retrograde cholangiopancreatography procedure was complicated by severe acute necrotizing pancreatitis with consequently splenic vein thrombosis, pseudocyst, and abscess formation in the pancreatic tail. Indication for total pancreatectomy was damage of the entire pancreas by necrotizing inflammation and portal vein thrombosis [10].

Another case of TPIAT as treatment for ampullary adenocarcinoma in the setting of pancreatic ductal disruption secondary to acute necrotizing pancreatitis has been reported by Iyegha et al. Pancreaticoduodenectomy was not a viable option in the setting of friable ductal tissue, which precluded pancreatic ductal anastomosis. [11]

Major differences between our case and those described in literature were three: the preoperative diagnosis of ampullary adenocarcinoma, the type of pathomorphological changes, and the islet transplantation. As mentioned above, in our case, an ampullary carcinoma was misidentified as a pancreatic cystic neoplasm. A diffuse pancreatic ductal dilatation in the organ, secondary to ampullary carcinoma, and the lack of a solid parenchyma were the factors which mainly impacted on the surgical strategy. Foci of chronic pancreatitis and endocrine tissue were scarcely spread out on the organ, but a pancreato-enteric anastomosis reconstruction was technically unfeasible.

Although TPIAT is progressively gaining acceptance as a treatment for refractory chronic pancreatitis and recurrent acute pancreatitis in order to control pain and improve diabetes outcomes [12], it is not a procedure routinely practised in our institution.

However, a risk of cancer cell contamination of the islet preparation and the subsequent risk of tumor recurrence should be taken into account [10].

## 4. Conclusion

Total pancreatectomy may be the gold standard treatment for patients with ampullary adenocarcinoma determining severe and permanent structural alterations of the entire pancreas which interfere with the pancreatic-enteric reconstruction. In this context, islet autotransplantation may support the postoperative glycemic control.

## Data Availability

The data used to support the findings of this study are included within the article.
